# Inhibition of Nuclear Factor-Kappa B Activation Decreases Survival of *Mycobacterium tuberculosis* in Human Macrophages

**DOI:** 10.1371/journal.pone.0061925

**Published:** 2013-04-25

**Authors:** Xiyuan Bai, Nicole E. Feldman, Kathryn Chmura, Alida R. Ovrutsky, Wen-Lin Su, Laura Griffin, Dohun Pyeon, Mischa T. McGibney, Matthew J. Strand, Mari Numata, Seiji Murakami, Loretta Gaido, Jennifer R. Honda, William H. Kinney, Rebecca E. Oberley-Deegan, Dennis R. Voelker, Diane J. Ordway, Edward D. Chan

**Affiliations:** 1 Department of Medicine, Denver Veterans Affairs Medical Center, Denver, Colorado, United States of America; 2 Departments of Medicine and Academic Affairs, National Jewish Health, Denver, Colorado, United States of America; 3 Department of Medicine, University of Colorado School of Medicine, Aurora, Colorado, United States of America; 4 Denver Health Medical Center, Denver, Colorado, United States of America; 5 Department of Microbiology, University of Colorado School of Medicine, Aurora, Colorado, United States of America; 6 Department of Medicine, Tri-Service General Hospital; National Defense Medical Center, Taipei, Taiwan; 7 Department of Microbiology, Immunology and Pathology, Colorado State University, Fort Collins, Colorado, United States of America; Fundação Oswaldo Cruz, Brazil

## Abstract

Nuclear factor-kappa B (NFκB) is a ubiquitous transcription factor that mediates pro-inflammatory responses required for host control of many microbial pathogens; on the other hand, NFκB has been implicated in the pathogenesis of other inflammatory and infectious diseases. Mice with genetic disruption of the p50 subunit of NFκB are more likely to succumb to *Mycobacterium tuberculosis* (*MTB*). However, the role of NFκB in host defense in humans is not fully understood. We sought to examine the role of NFκB activation in the immune response of human macrophages to *MTB*. Targeted pharmacologic inhibition of NFκB activation using BAY 11-7082 (BAY, an inhibitor of IκBα kinase) or an adenovirus construct with a dominant-negative IκBα significantly decreased the number of viable intracellular mycobacteria recovered from THP-1 macrophages four and eight days after infection. The results with BAY were confirmed in primary human monocyte-derived macrophages and alveolar macrophages. NFκB inhibition was associated with increased macrophage apoptosis and autophagy, which are well-established killing mechanisms of intracellular *MTB*. Inhibition of the executioner protease caspase-3 or of the autophagic pathway significantly abrogated the effects of BAY. We conclude that NFκB inhibition decreases viability of intracellular *MTB* in human macrophages via induction of apoptosis and autophagy.

## Introduction

Tuberculosis (TB) remains problematic throughout much of the world due, in large part, to burgeoning drug resistance and human immunodeficiency virus (HIV) co-infection [1]–[3]. In addition to these critical barriers to TB control, *Mycobacterium tuberculosis* (*MTB*) has been highly successful in evading host immune mechanisms and surviving within phagocytes [4]–[7]. Gaining a better understanding of host-pathogen interactions will enable the development of novel therapeutics to treat drug resistant cases and shorten current treatment time.

The seminal work of Lurie demonstrated that innate immunity is particularly important in the protective response against pulmonary TB [8] and is largely mediated by a number of phagocytic receptors and pattern-recognition receptors such as complement receptors and Toll-like receptors (TLRs) on macrophages [9]–[17]. Upon engagement of TLRs by microbial ligands, the I-kappa B-alpha (IκBα) kinase-nuclear factor-kappa B (IKK-NFκB) signaling cascade is activated, resulting in NFκB-mediated transcription of pro-inflammatory genes [18]–[21]. By inducing an inflammatory response, NFκB activation has been shown to enhance immunity against certain microbial pathogens [22], [23]. However, specific NFκB-mediated pathways may be exploited by bacterial pathogens to promote survival; *e.g.*, *Escherichia coli* and *Chlamydophila pneumoniae* activate NFκB to prevent apoptosis of host cells [22], while *Shigella flexneri* and *Helicobacter pylori* induce host NFκB to enhance tissue invasion [24], [25].

We examined how inhibition of NFκB activation impacts the viability of intracellular *MTB* in human macrophages including differentiated THP-1 monocytes, primary monocyte-derived macrophages (MDM), and primary alveolar macrophages (AM). We found that inhibiting NFκB activation reduced the viability of intracellular *MTB* in all three types of human macrophages. We also examined the impact of inhibiting NFκB activation on apoptosis and autophagy [26]–[28], two cellular processes known to facilitate mycobacterial killing [4], [6], [29]–[33]. We discovered that inhibition of NFκB activation reduces intracellular survival of *MTB* by enhancing both host-protective apoptosis and autophagy of the infected macrophages.

## Materials and Methods

### Materials

The human promonocytic cell line THP-1 (TIB-202) and *MTB* H37Rv (27294) were obtained from the American Type Culture Collection (Manassas, VA). The following reagents were purchased: RPMI cell culture medium (Cambrex, East Rutherford, NJ), FBS heat-inactivated at 56°C for 1 hr (Atlanta Biologicals, Norcross, GA), BAY 11-7082 (BAY) – a specific IKK inhibitor (Biomol Research Laboratories, Plymouth Meeting, PA), TNFα ELISA kit (Life Technologies, Grand Island, NY), reagents for Middlebrook 7H10 solid agar medium (Difco, Detroit, MI), ^32^γ-ATP (>3000 Ci/mmol) (NEN Research Products DuPont, Wilmington, DE), and dimethyl sulfoxide (DMSO), phorbol myristate acetate (PMA), and 3-methyladenine (3-MA) (Sigma, St. Louis, MO). The polyclonal rabbit antibody directed against microtubule-associated protein light chain 3 (LC3), cytochrome c antibody, and β-actin antibody were purchased from Cell Signaling Technology (Danvers, MA). The caspase-3 inhibitor benzyloxycarbonyl-Asp-Glu-Val-Asp-fluoromethylketone (z-DEVD-fmk) and ELISA kits for detecting active caspase-3 (Human Active Caspase-3 Immunoassay) and IL-8 were purchased from R & D Systems, Inc. (Minneapolis, MN). The EIA-lacking adenovirus vector (AdV) cloned to a mutant IκBα in which serine 32 and 36 residues were mutated to alanine (AdV-S32/36A-IκBα) and an AdV-green fluorescent protein (AdV-GFP) construct were gifts of Drs. Adela Cota-Gomez and Sonia Flores of University of Colorado Anschutz Medical Center.

### Culture of *MTB* stock

*MTB* was grown to log phase at 37°C in Difco Middlebrook 7H9 medium (Becton Dickinson, MD), enriched with 10% stock ADC Enrichment (Remel, Lenexa, KS) containing 5% (w/v) BSA fraction V, 2% (w/v) glucose, 0.87% (w/v) NaCl, and 0.004% (w/v) catalase. Tween 80 (0.05%, v/v) and glycerol (0.2%, v/v) were also added to the growth medium. After culture of the mycobacteria under aeration, the culture was diluted to a concentration of 1.0 McFarland standard (equivalent to 10^8^ bacilli/mL) and stored at −80°C.

### Differentiation of THP-1 cells

THP-1 cells were cultured in RPMI 1640 medium supplemented with 10% FBS and 2 mM glutamine and were maintained between 2 and 10×10^5^ cells/mL. Prior to experimentation, THP-1 cells were stimulated with 15 ng/mL PMA for 2 to 3 days to allow differentiation into macrophages. The PMA-containing medium was removed and replaced with fresh medium just prior to exposing the cells to the experimental conditions.

### Isolation of human monocyte-derived macrophages

Nine healthy, non-smoking volunteers, 21 to 65 years of age, were recruited for blood donation after National Jewish Health Institutional Review Board (NJH-IRB) approval and written informed consent was obtained from each enrolled subject. Human monocytes were isolated from 50 mL of heparinized blood and process for differentiation into macrophages as we previously described [34]. In brief, the samples were centrifuged at 400×*g* at room temperature for 25 min. The white buffy coat layer was removed, washed, counted, and resuspended in RPMI medium containing 10% FBS to a concentration of 4× 10^6^ cells/mL. One-half mL of the cell suspension (2×10^6^ cells/0.5 mL) was added to each well of a 24-well polystyrene plate, estimated to yield about 2×10^5^ MDM assuming 10% of peripheral white blood cells are monocytes. The cells were incubated at 37°C in a humidified 5% CO_2_ incubator for 10–14 days, and the media were replaced on days 2, 5, 7, 9 and 12, resulting in the selection of MDM.

### Isolation of alveolar macrophages

Nine healthy, non-smoking volunteers, 21 to 65 years of age, were recruited for bronchoalveolar lavage to obtain AM after NJH-IRB approval and written informed consent was obtained from each enrolled subject. All bronchoscopies were performed by EDC. The bronchoscope was wedged in a segment of the right middle lobe and four-60 mL aliquots of sterile normal saline were instilled and sequentially aspirated back. The volume of lavage recovered was typically 60 to 70% of the amount instilled. The bronchoalveolar lavage fluid was centrifuged at 200×*g* for 10 min at 4°C. Cell pellets were washed with PBS and resuspended in 10 mL RPMI medium containing 10% FBS and 100 U/mL penicillin G. Cells were counted using a hemocytometer and the volume of medium was adjusted to give a concentration of 1.0×10^6^ cells/mL. One-quarter mL (2.5×10^5^ cells) of this suspension plus 250 µL of RPMI medium was added to each well of a 24-well plate and incubated at 37°C in a humidified 5% CO_2_ incubator. After 24 hours of incubation, the medium was replaced with fresh antibiotic-free RPMI medium containing 10% FBS and incubated overnight. Prior to infection with *MTB*, the medium was replaced a second time with antibiotic-free RPMI medium to remove any trace of penicillin G.

### Infection of macrophages with *MTB*

Macrophages were infected with *MTB* H37Rv at a multiplicity of infection (MOI) of 10 bacilli to 1 macrophage as previously described [35]. In conditions where NFκB was inhibited, the cells were pre-incubated with 5 µM BAY or 0.1% (v/v) DMSO for 1 hr prior to infection.

### Culture of cell-associated *MTB*

For cells infected for 1 hr (Day 0), the supernatants were collected after 1 hr of infection and the cells washed twice with a 1∶1 solution of RPMI:1× PBS. Adherent cells were lysed with 250 µL of a 0.25% SDS solution per well, followed by addition of 250 µL of 7H9 plating broth. Serial dilutions of cell lysates were prepared and then 5 µL of each dilution was plated on Middlebrook 7H10 agar. For Day 4 and 8 infections, the cells were washed twice after the initial 1 hr of infection and replaced with fresh RPMI medium containing 10% FBS±5 µM BAY; the medium was not changed until 4 or 8 days after infection. After 4 and 8 days of infection, the supernatants were centrifuged to recover any non-adherent macrophages; these macrophages were lysed together with the adherent macrophages and *MTB* cultured as described above. We considered it important to culture both adherent and non-adherent macrophages to accurately recover all cell-associated *MTB*. The cell-free supernatants were collected for cytokine analysis.

### Construction and detection of *MTB* H37Rv-GFP

*MTB* H37Rv was transformed with the plasmid pBCM. In brief, the GFP fragment recovered from digestion of plasmid pFPCA1 [36] with BamHI/EcoRI was cloned into pMV261 to make pBCM. pMV261 (Addgene) is a replicative vector that contains a kanamycin resistance gene, an *E.coli* origin of replication (*oriE*), a mycobacterial plasmid DNA origin of replication (*oriM*), an expression cassette containing a mycobacterial promoter (Hsp60), a multiple cloning site, and a transcriptional terminator. After transformation of *MTB* H37Rv with pBCM by electroporation, positive clones were selected on Middiebrook 7H10 agar which contains 50 µg/mL of kanamycin and GFP-labeled *MTB* detected by fluorescent microscopy.

THP-1 cells (2×10^5^ cells/200 µL/well) were plated in black, clear-bottom 96-well microtiter plates in order to minimize background fluorescence (Black Viewplates, Packard Instrument Company, Meriden, Conn). Following overnight differentiation with PMA, the cells were incubated with 5 µM BAY for 1 hr and then infected with *MTB* H37Rv-GFP at a MOI of 10 for 1 hr. For the 1 hr time point, the medium was removed, 4% paraformaldehyde was added for 1 hr to fix the cells, washed three times with PBS, and fluorescence was measured using a Cytofluor II microplate fluorometer in the bottom-reading mode with excitation and emission wavelengths of 485 nm and 508 nm, respectively. For the 4 and 8 day time points, the medium was changed to fresh medium containing 5 µM BAY, and the *MTB*-infected cells were incubated at 37°C in 5% CO_2_ for an additional 4 and 8 days before the same preparations were made to measure fluorescence. The mean fluorescent value of three wells containing uninfected THP-1 cells was subtracted from the measured fluorescence of all test wells.

### Electrophoretic mobility shift assay

NFκB activation was measured by electrophoretic mobility shift assay (EMSA). The human macrophages were either untreated, or pre-treated with 5 µM BAY or 0.1% DMSO vehicle for 1 hr, followed by infection with *MTB* H37Rv at a MOI of 10 for 30 min, 1 hr, 3 hrs, or 6 hrs. The methods used for the nuclear protein extraction, end-labeling of a double stranded oligonucleotide (Promega, Madison, WI) containing a consensus sequence for NFκB with ^32^P-γ-ATP, and binding reaction of the nuclear proteins with the labeled oligonucleotide are as previously published [37], [38]. One additional step was that the extracted nuclear protein was filtered through a 0.2 µm syringe filter to remove any *MTB*.

### Cytokine measurements

The concentrations of TNFα and IL-8 in the culture supernatants were determined by ELISA according to manufacturer's instructions. The level of IFNγ was determined by electrochemiluminescence as previously described [35], [39].

### Apoptosis assay

Apoptosis was determined by terminal deoxynucleotidyl transferase dUTP nick end labeling (TUNEL). Cell suspensions (0.5 mL) were grown on 4-chamber well slides [34], pre-incubated with BAY and then infected with *MTB* H37Rv at a MOI of 10. The medium was removed at 4 and 8 days following infection and the cells were fixed in 4% paraformaldehyde solution (pH 7.4). The cells were then stained with an Apoptosis In Situ Detection Kit (Roche Diagnostic, Indiannapolis, IN) according to the manufacturer's instructions and as previously reported [35]. Enumeration of apoptotic cells was performed by light microscopy, counting ∼5000 cells per condition per experiment.

### Western blotting

Expression of the autophagosome-associated LC3-II and its precursor LC3-I as well as cytochrome c were detected by western blotting using methods previously described [35]. In brief, after SDS-PAGE of nuclear-free whole cell lysates (20 µg protein per condition), the proteins were transferred onto a nitrocellulose membrane (ISC BioExpress, Kaysville, UT), blocked in 10% (w/v) non-fat dry milk at 4°C overnight with shaking, and immunoblotted with 1∶1000 solution of anti-LC3B antibody in 5% BSA (w/v) or anti-cytochrome c antibody in 5% (w/v) non-fat dry milk for 1 hr at room temperature shaking. After incubation with horseradish peroxidase-conjugated secondary anti-rabbit antibody (1∶2000) in 5% (w/v) non-fat dry milk for 1 hr at room temperature, bound antibodies were detected by enhanced chemoluminescence.

### Direct fluorescence to examine autophagosome formation

Autophagosomes can be directly visualized as cytoplasmic granules (punctae) that stain positive for LC3-II, a lipoprotein specific for autophagosomal membranes [40]. To quantify LC3-II-positive punctae in *MTB*-infected THP-1 cells with NFκB inhibition, recombinant GFP-LC3 lentivirus was generated in the HEK 293 cell line (Cell Biolabs, Inc, San Diego, CA). Lentiviruses were prepared by co-transfecting packaging plasmids pVSV-g (Dr. Jerome Schaack, University of Colorado School of Medicine) and pCMV-HIVdeltaR8.2 (Addgene, plasmid #12263) using an established protocol [41]. The GFP-LC3 expression plasmid, pCDH-GFP-LC3, was generated by subcloning the GFP-LC3 gene from the pEGFP-C3 vector backbone (Addgene) into the pCDH-puro lentivirus expression vector. GFP-LC3 lentivirus was transduced into THP-1 cells at a multiplicity of 10 lentiviruses to one THP-1 cell in the presence of 8 µg/mL Polybrene; GFP lentivirus served as a positive control and empty vector lentivirus as a negative control. To quantify autophagosome formation by fluorescence, GFP-LC3 lentivirus-transduced cells were plated on chamber slides at 10^5^ cells per well, and allowed to differentiate overnight with 15 ng/mL PMA at 37°C in 5% CO_2_. The next day, the PMA-containing medium was replaced with fresh THP-1 medium (RPMI+10% FBS+1% glutamine). BAY was added at a concentration of 5 µM per well and was incubated for 1 hr prior to infection with *MTB*. Cells were infected with *MTB* H37Rv at a MOI of 10 for 24 hrs and fixed in a 4% paraformaldehyde solution for 1 hr at room temperature. After rinsing with PBS, the cells were then stained with ProLong Gold Antifade reagent with DAPI (Invitrogen) and placed in the dark at 4°C until use. For quantifying the number of autophagosomes per cell, the number of GFP-LC3 punctae were counted from at least 100 cells/well in duplicate wells for each condition. All the cellular images were obtained using an inverted confocal fluorescence microscope (Nikon, Carl Zeiss Axiovert 200 M) and the data analysis using correlation analysis tool of Slidebook program (Slidebook 4.2).

### Statistical analysis

Replicate experiments were independent, and where appropriate, summary results are presented as means ± SEM. Differences were considered significant for p<0.05, and all reported p-values used a two-sided test. For most experiments, group means were compared by ANOVA using Fisher's least significance difference procedure. The AM and MDM cytokine data were analyzed as follows to account for repeated measures over treatment and time within subjects. Each outcome variable was fit with a linear mixed model, using predictor variables of *MTB* (Y, N), BAY (Y, N), Time (1, 24, 96, 192 hours), and all interactions of these, up to the 3-way interaction. Repeated measures on subjects over treatment (4 combinations of *MTB*–Y/N and BAY–Y/N) and time were accounted for using a direct (Kronecker) product structure for all outcome variables except for those noted below. The “unstructured” covariance structure was used for repeated measures involving time, while the compound symmetry structure was used for those involving treatment; the direct product structure allows these to be modeled simultaneously in one combined covariance structure. TNFα and IFNγ produced by MDM used a simpler compound symmetric covariance structure since for these outcome variables the models did not converge using the direct product covariance structure.

Outcome variables were log transformed for analysis when necessary (AM-TNFα, THP-1-TNFα, and AM-IFNγ), but results for all outcome variables are presented on the original scale; those analyzed on the log scale were inverted back for presentation.

Specific pairwise comparisons of interest were between times for the *MTB* only treatment, and within times between the *MTB* only and *MTB* + BAY treatments. Such comparisons were only considered when at least one predictor variable (main effect or interaction) in the related model was significant.

## Results

### *MTB*-induced NFκB activation is inhibited by BAY

**Figure 1:**
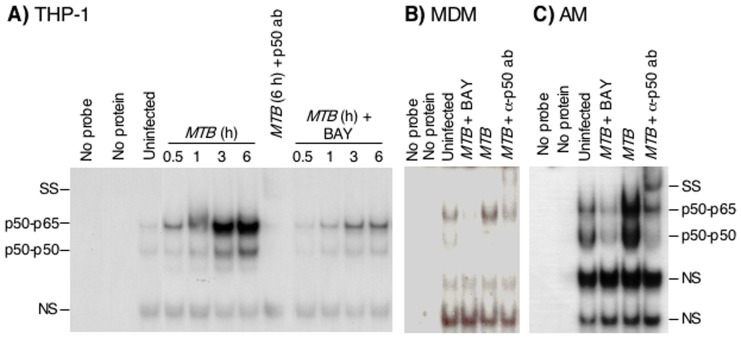
*MTB* H37Rv induces NFκB binding to its *cis*-regulatory element. (**A**) THP-1 cells were pre-treated with 0.1% (v/v) DMSO or 5 µM BAY for 1 hr, followed by infection with *MTB* H37Rv at the indicated times. An EMSA was performed with an oligonucleotide that corresponds to the consensus binding sequence for NFκB. Data shown are representative of three independent experiments. (**B**) Primary human MDM or (**C**) AM were pre-incubated with 0.1% DMSO or 5 µM BAY for 1 hr, then infected with *MTB* for 3 hrs, and followed by an EMSA to assay for NFκB binding. Data shown are representative of two independent experiments for MDM and AM. NS = non-specific band. SS = supershift band.

*MTB* induced NFκB activation in THP-1 cells, particularly after 3 and 6 hrs of infection (**Figure 1A**). In the presence of 5 µM BAY, NFκB activation was strongly inhibited (**Figure 1A**). Co-incubation of the nuclear extract with anti-p50-NFκB antibody further retarded the migration of NFκB complexes, previously identified to be p50/p50 and p50/p65 [38]. There was no further reduction in NFκB activation following administration of 7.5 µM or 10 µM BAY (data not shown).

We pre-incubated primary human MDM and AM with 5 µM BAY or 0.1% (v/v) DMSO vehicle for 1 hr, followed by infection with *MTB* H37Rv for an additional 3 hrs. Similar to THP-1 cells, *MTB* infection of primary MDM and AM induced NFκB activation, and this activation was inhibited by BAY (**Figure 1B** **/C**).

### Inhibition of NFκB significantly reduces the viability of intracellular *MTB*

**Figure 2:**
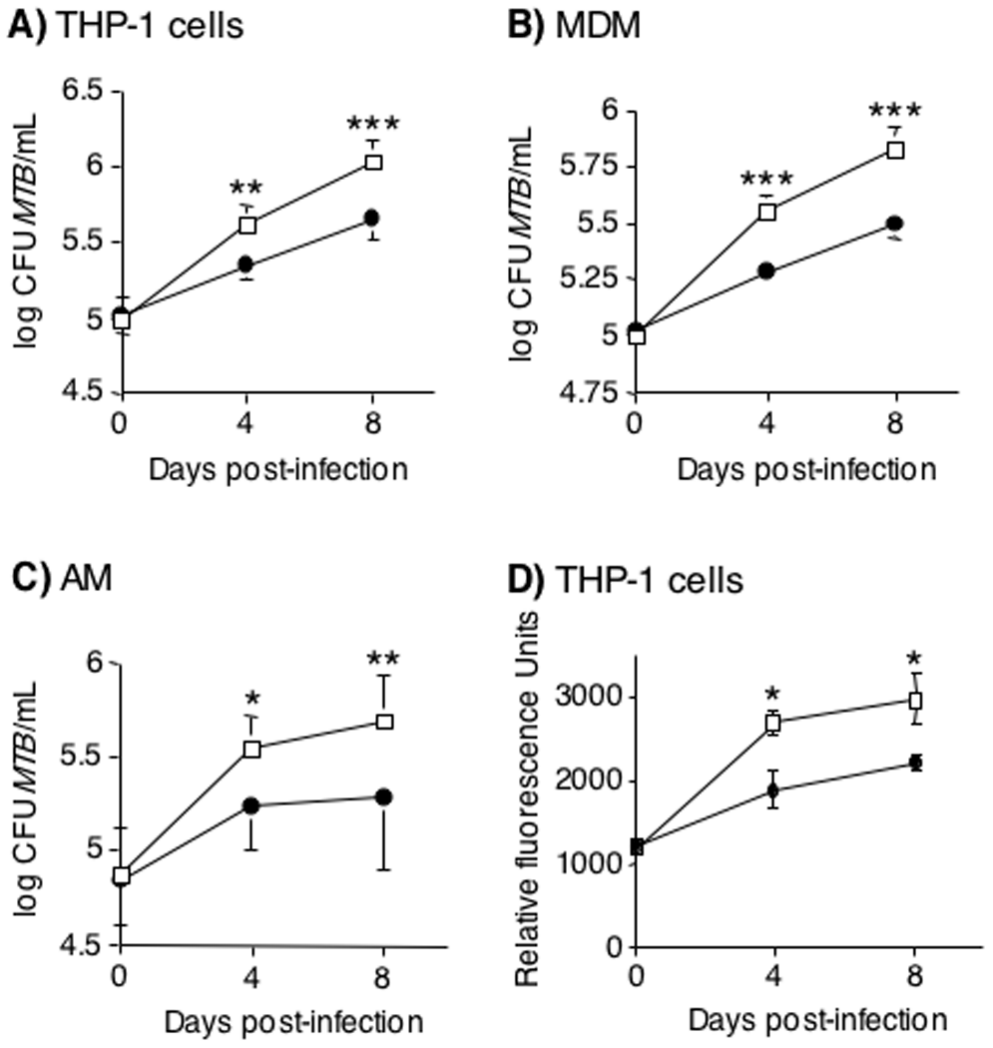
Inhibition of NFκB activation by BAY 11-7082 reduces the viability of intracellular *MTB* in human macrophages. (A) THP-1 cells, (B) MDM, or (C) AM were pretreated with 0.1% (v/v) DMSO (open squares) or 5 µM BAY (closed circles) for 1 hr, followed by infection with *MTB* H37Rv. One hr, 4 days, or 8 days after infection, the cells were lysed and cultured for *MTB*. (D) Differentiated THP-1 cells were pretreated with 0.1% (v/v) DMSO (open squares) or 5 µM BAY (closed circles) for one 1 hr, followed by infection with *MTB*-H37Rv-GFP. One hr, 4 days, or 8 days after infection, fluorescent intensity was measured by Cytofluor II microplate fluorometer. Data shown as mean ± SEM. n = 4 for THP-1 cells in (A) and n = 2 for THP-1 cells in (D), n = 7 volunteers for MDM, n = 9 volunteers for AM. *p<0.05, **p<0.01, ***p<0.001.

Differentiated THP-1 cells, MDM, and AM were used to determine the impact of NFκB inhibition on intracellular recovery of viable *MTB*. The mean number of intracellular *MTB* isolated 1 hr following infection (Day 0) was similar between the control cells and cells pre-treated with BAY, indicating that inhibition of NFκB did not impact the phagocytosis of *MTB* by the macrophages (**Figure 2A,B,C**). However, inhibition of NFκB activation significantly reduced the number of intracellular *MTB* recovered 4 days after infection from THP-1 cells by 64%, from MDM by 67%, and from AM by 63% (**Figure 2A,B,C**). By 8 days after infection, BAY significantly reduced the number of viable intracellular *MTB* by 66% in THP-1 cells, 63% in MDM, and 71% in AM (**Figure 2A,B,C**). There was no significant difference in the number of CFU recovered with higher concentrations of BAY (7.5 and 10 µM) compared to 5 µM BAY (data not shown). In the absence of macrophages, 5 µM or 10 µM BAY had no effect on viability of *MTB* H37Rv compared to *MTB* cultured with 0.1% DMSO vehicle (**Figure S1**).

Trypan blue staining was performed on control THP-1 cells and cells incubated for 4 days with 5 µM BAY or BAY + *MTB*. Approximately 11% of the cells infected with *MTB* alone were non-viable whereas ∼20% of the cells were non-viable after incubation with both *MTB* and BAY (**Figure S2**).

While we attempted to recover any non-adherent cells at the Day 4 and Day 8 time points since it would be important to include any live *MTB* in these cells, some cells may have been lost in the recovery process. To validate the CFU data, we infected THP-1 cells with *MTB* H37Rv-GFP in the presence or absence of BAY at the aforementioned times of infection. After the indicated times, the cells were washed, fixed, and fluorescence intensity measured. As shown in **Figure 2D**, the fluorescent intensity of the BAY-treated THP-1 cells was significantly less than untreated *MTB*-infected cells at 4 and 8 days after infection. Moreover, the relative slope of the fluorescent curves roughly matched those of the CFU growth curves.

**Figure 3:**
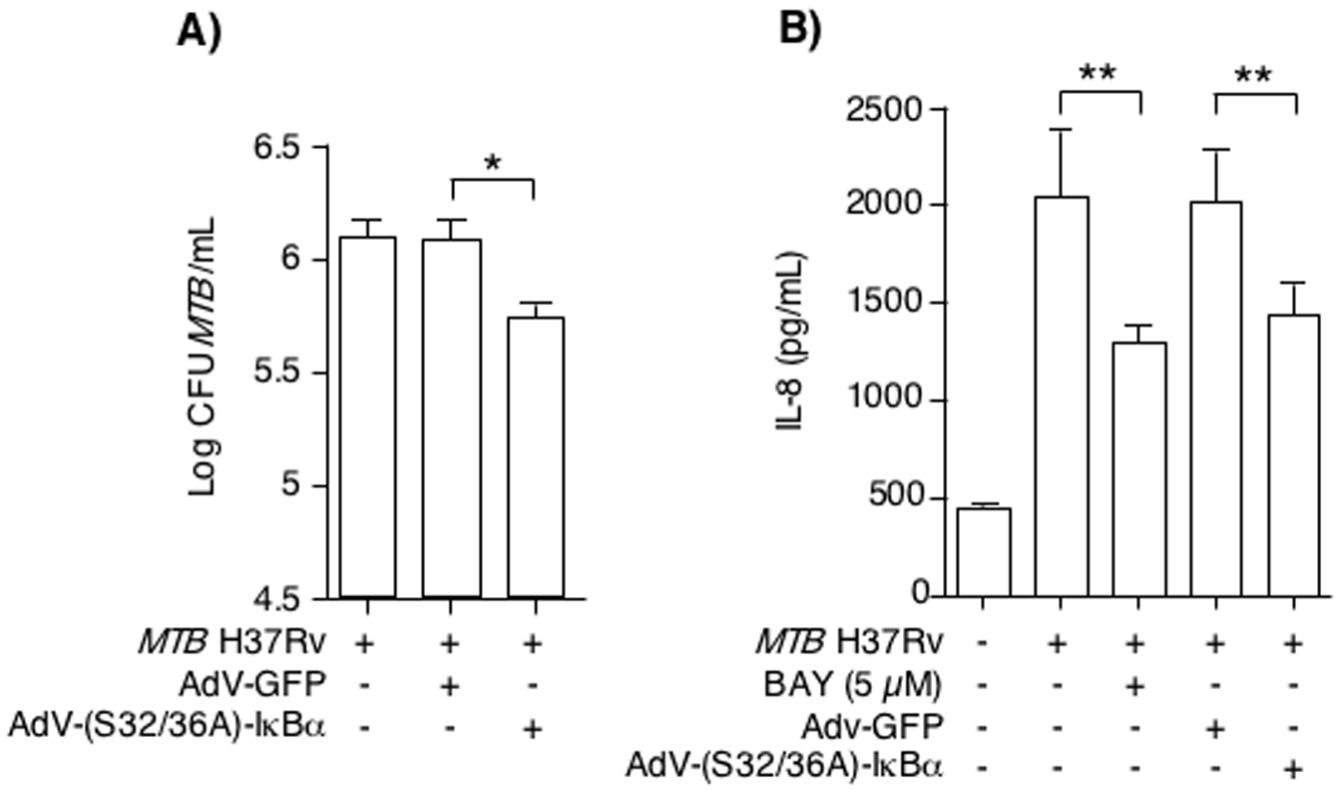
Inhibition of NFκB activation using a dominant-negative to IκBα. (**A**) THP-1 cells were transduced with or without AdV-GFP or AdV-S32/36A-IκBα at a MOI of 30∶1 for 5 hrs and then infected with *MTB* H37Rv. After 4 days of infection, the cells were lysed and cell-associated *MTB* quantified. (**B**) Wildtype THP-1 cells with or without pre-treatment with 5 µM BAY were infected with *MTB* H37Rv. Other THP-1 cells were transduced with AdV-GFP or AdV-S32/36A-IκBα at a MOI of 30∶1 for 5 hrs and then infected with *MTB* H37Rv. After 24 hrs of infection, the supernatants were measured for IL-8 by ELISA. Data are means ± SEM of two independent experiments performed in duplicates. *p<0.05, ** p<0.01.

While BAY does not inhibit other signaling pathways such as the mitogen-activated protein kinases [42], [43], we validated our findings by transducing THP-1 cells with an EIA-deficient adenovirus (AdV) construct containing a mutant IκBα (AdV-S32/36A-IκBα) [44], [45]. Due to lack of EIA, this attenuated AdV-S32/36A-IκBα vector is unable to replicate but because the mutated IκBα cannot be phosphorylated, this dominant-negative IκBα remains a potent inhibitor of NFκB activation. We have previously used this construct to effectively inhibit *MTB* lipoarabinomannan activation of NFκB [38]. THP-1 cells were transduced with AdV-GFP (control vector) or AdV-S32/36A-IκBα for 6 hrs, infected with *MTB* at a MOI of 10 for 4 days, and cell-associated *MTB* were quantified. As shown in **Figure 3A**, AdV-GFP-infected cells had similar numbers of *MTB* compared to *MTB* infection of wildtype THP-1 cells; in contrast, the AdV-S32/36A-IκBα-infected cells had significantly reduced number of cell-associated *MTB*, consistent with the results of NFκB inhibition by BAY. To confirm AdV-S32/36A-IκBα is biologically active in THP-1 cells, we determined whether *MTB*-induction of IL-8 could be inhibited since induction of IL-8 is critically dependent on NFκB [46], [47]. THP-1 cells infected with *MTB* for 24 hrs induced IL-8 but co-incubation with BAY significantly inhibited IL-8 expression (**Figure 3B**). THP-1 cells transduced with Adv-GFP and infected with *MTB* also had robust induction of IL-8; in contrast, THP-1 cells transduced with AdV-S32/36A-IκBα and infected with *MTB* had significantly lower amounts of *MTB*-induced IL-8 compared to AdV-GFP transduced cells (**Figure 3B**).

### Inhibition of NFκB increases apoptosis of macrophages

**Figure 4:**
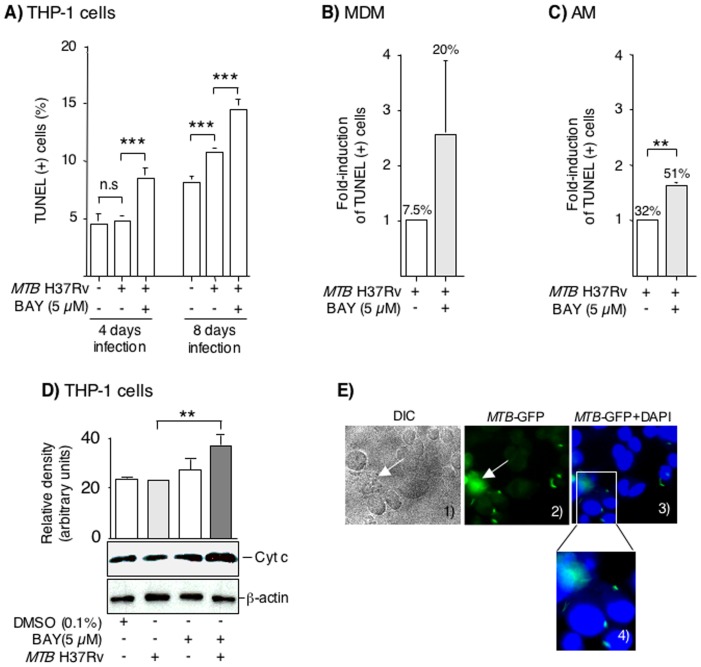
Inhibition of NFκB activation increases apoptosis of infected macrophages. (**A**) THP-1 cells were infected with *MTB* H37Rv for 4 and 8 days with or without BAY, and apoptosis measured by TUNEL. Data for THP-1 cells are the mean ± SEM of four independent experiments. (**B**) Primary human MDM and (**C**) AM were infected with *MTB* with or without BAY, cultured for 4 days, and apoptosis measured by TUNEL. The percentage (%) numbers above the bars indicate the % cells with positive TUNEL stain. Data for MDM and AM are the mean ± SEM of three independent experiments. n.s. = not significant, **p<0.01, ***p<0.001. (**D**) THP-1 cells were infected with *MTB*, 5 µM BAY 11-7082, or both. After 48 hrs, nuclear-free whole cell lysates isolated, and western blot performed for cytochrome c. The membranes were also immunoblotted for β-actin. The bar graph above the immunoblot represent the mean relative density measurements for cytochrome c bands normalized for the densities of the corresponding β-actin band. The data shown are representative of two independent experiments. **p<0.01. (**E**) THP-1 cells were infected with *MTB* H37Rv-GFP for 1 hr, stained with DAPI, and viewed under both differential interference contrast (DIC) and fluorescent imaging under 630× magnification (panels 1–3). An area of panel 3 was magnified further on the computer screen (panel 4). Data shown are representative of two independent experiments.

Apoptosis contributes to the intracellular killing of mycobacteria [30], [48]–[51]; thus, the effect of NFκB inhibition on apoptosis of infected macrophages was evaluated. Following 4 days of incubation, there was no difference in extent of TUNEL staining between uninfected THP-1 cells and THP-1 cells infected with *MTB* (**Figure 4A**). However, there was a significant increase in apoptosis at 4 days (relative increase in apoptosis of 79%) in BAY-treated, *MTB*-infected THP-1 cells (**Figure 4A**). After 8 days of infection, there was a modest but significant increase in apoptosis with *MTB* infection alone compared to uninfected cells (relative increase in apoptosis of 34%); in the presence of BAY, there was a further significant increase in apoptosis of *MTB*-infected THP-1 cells (**Figure 4A**).

BAY alone was also found to modestly increase apoptosis of THP-1 cells in the absence of *MTB* at 24 hrs; *i.e.*, the level of TUNEL positivity in THP-1 cells alone was 2.45%; with the addition of 5 µM BAY, the percent of TUNEL positivity increased to 5.37%. These results suggest that basally, low levels of NFκB activation may prevent spontaneous apoptosis in differentiated THP-1 cells.

We next quantified TUNEL positivity in the *MTB*-infected MDM and AM in the presence or absence of BAY. *MTB*-infected MDM and AM showed increased apoptosis after 4 days of infection in the presence of BAY, with relative increases of 170% and 60%, respectively (**Figure 4B,C**).

To corroborate that apoptosis is induced by *MTB* and BAY, we analyzed the level of cytochrome c, an essential component of the intrinsic apoptotic pathway, in THP-1 cells infected with *MTB* with and without BAY after 48 hrs. *MTB* alone had little or no induction of cytochrome c as assessed by western blotting (**Figure 4D**). BAY 11-7082 treatment alone had a modest induction of cytochrome c whereas culture of THP-1 cells with both *MTB* and BAY showed a significant induction of cytochrome c (**Figure 4D**).

While the *absolute* change in apoptosis was small, there was a large decrease in bacterial recovery from macrophages. This phenomenon can occur when killing mechanisms other than apoptosis are involved but also when macrophages are non-uniformly infected with *MTB*. To assess the latter, THP-1 cells were infected with *MTB* H37Rv-GFP for 1 hr and visualized by fluorescent microscopy. Shown in lower magnification is a representative differential interference contrast (DIC) image (**Figure 4E** **, panel 1**). In a representative fluorescent image, one macrophage is heavily infected (**arrows in panels 1 and 2 of** **Figure 4E**) while others were much less burdened or not infected (**Figure 4E** **, panel 2**). The cells were also stained with DAPI and the merged images are shown in **Figure 4E** **, panel 3** and further magnified in **Figure 4E** **, panel 4**. Similar findings were also seen with the infected MDM and AM with Auramine acid-fast staining (data not shown).

### Caspase-3 inhibition abrogates BAY-induced apoptosis

**Figure 5:**
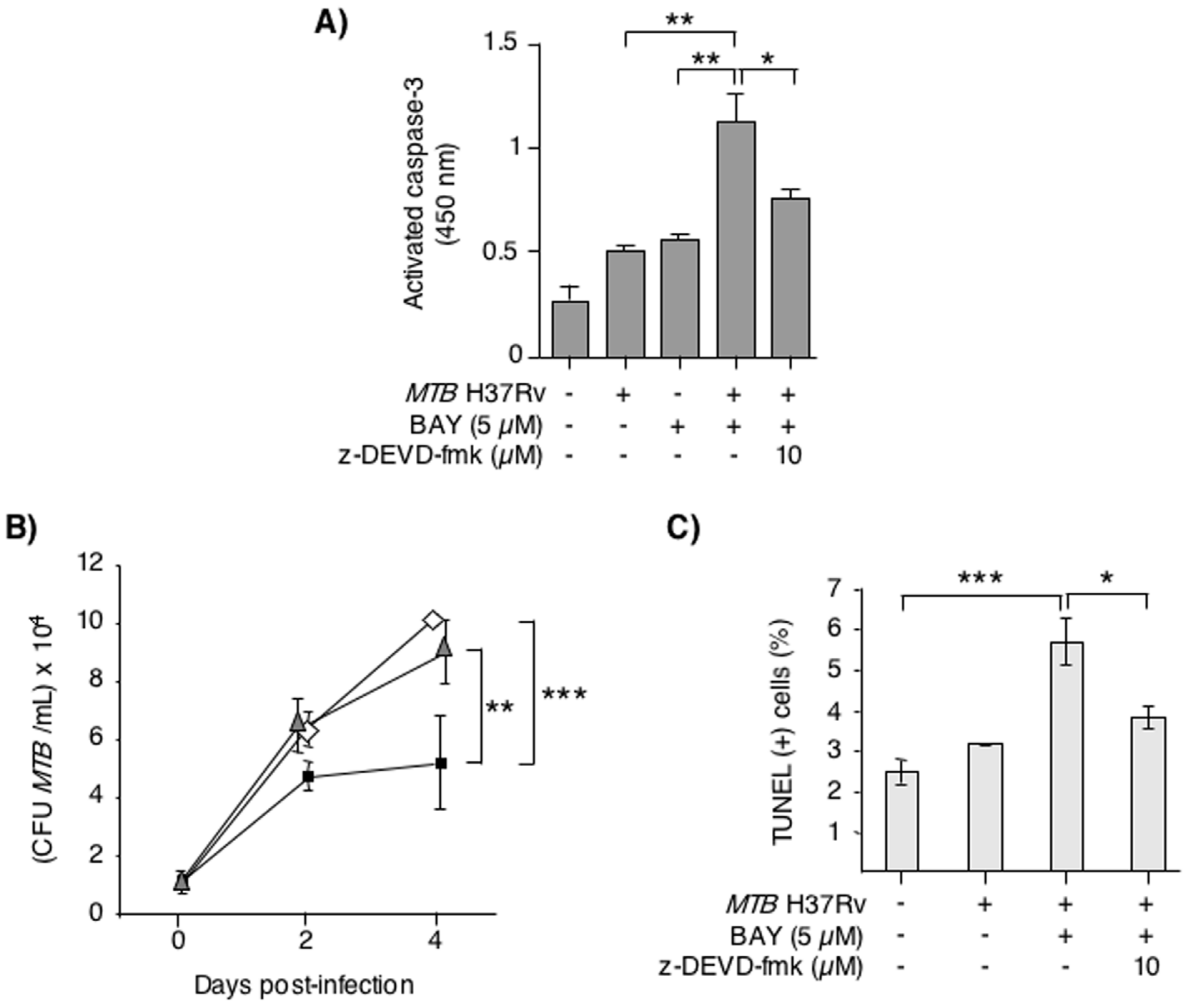
Inhibition of caspase-3 activation abrogates the effects of BAY. (**A**) THP-1 cells were cultured with 5 µM BAY, *MTB*, or *MTB*+BAY with or without 10 µM of the caspase-3 inhibitor z-DEVD-fmk for 48 hrs. After the indicated time, the cells were lysed and activated caspase-3 quantified by ELISA. Data shown are the mean ± SEM of two independent experiments performed in duplicates. *p<0.05, **p<0.01. (**B**) THP-1 cells were infected with *MTB* H37Rv alone (open diamonds), *MTB*+BAY (closed squares), or *MTB*+BAY+z-DEVD-fmk (semi-closed triangles). One hr, 2 days, and 4 days after infection, THP-1 cells were lysed and cultured for *MTB*. (**C**) The same treatment conditions as in (A) were repeated with THP-1 cells for 2 days followed by measurement of apoptosis using TUNEL staining. Data shown are the mean ± SEM of two independent experiments. *p<0.05, **p<0.01 and ***p<0.001.

To determine whether BAY-induced apoptosis impacted viability of intracellular *MTB*, we analyzed the effect of inhibiting apoptosis using a specific caspase-3 inhibitor (z-DEVD-fmk). Caspase-3 is an executioner caspase for both the intrinsic and extrinsic pathways of classical apoptosis. THP-1 cells infected with *MTB* or cultured with BAY for 48 hrs showed a modest induction of activated caspase-3 whereas cells cultured with both *MTB* and BAY showed robust caspase-3 activation (**Figure 5A**). Co-culture with 10 µM of the caspase-3 inhibitor (z-DEVD-fmk) showed a significant abrogation of *MTB*+BAY activation of caspase-3 (**Figure 5A**). THP-1 cells were infected with *MTB* alone, *MTB*+5 µM BAY, or *MTB*+BAY and 10 µM z-DEVD-fmk. At 1 hr, 2 days, and 4 days after infection, cells were lysed and lysates cultured for *MTB*. As seen previously, compared to *MTB* alone, addition of BAY significantly reduced the number of *MTB* in THP-1 cells (**Figure 5B**). With caspase-3 inhibition, BAY-mediated decrease in CFU was significantly abrogated (**Figure 5B**).

To confirm that caspase-3 inhibition reduces the induction of apoptosis by *MTB* and BAY, infected THP-1 cells were incubated with BAY in the presence or absence of the specific caspase-3 inhibitor, z-DEVD-fmk. Increased apoptosis was observed in BAY+*MTB*-infected cells after 48 hrs; in the presence of z-DEVD-fmk, *MTB* plus BAY induced apoptosis was significantly suppressed (**Figure 5C**). There was no significant difference between the effects of 10 µM and 20 µM of z-DEVD-fmk in regard to both intracellular number of *MTB* and apoptosis (data not shown).

### Inhibition of NFκB induces autophagy in *MTB*-infected THP-1 cells

Autophagy is a cellular process by which cytoplasmic organelles and molecules are catabolized, and is increasingly recognized as an effector mechanism by which host cells kill intracellular *MTB* and other intracellular pathogens [4], [6], [52]. With initiation of autophagy, an LC3-II-mediated double membrane forms around the cytoplasmic or intraphagosomal organisms, forming autophagosomes that more readily fuses with lysosomes, resulting in the killing of the organisms by lysosomal machinery [40].

Immediately following synthesis of LC3, its C-terminal fragment is cleaved to yield a cytosolic form of LC3 termed LC3-I. During induction of autophagy, LC3-I is conjugated with phosphatidylethanolamine (PE) to form LC3-II, which binds to both the outer and inner autophagosomal membranes [53]. Thus, LC3-II is a highly specific marker of autophagosome formation [6]. Although LC3-II has a higher molecular weight than LC3-I, it runs lower on SDS-PAGE due to the physical properties of the lipid attachment. Thus, we determined the effects of NFκB inhibition and *MTB* infection on the levels of the autophagic marker LC3-II.

**Figure 6:**
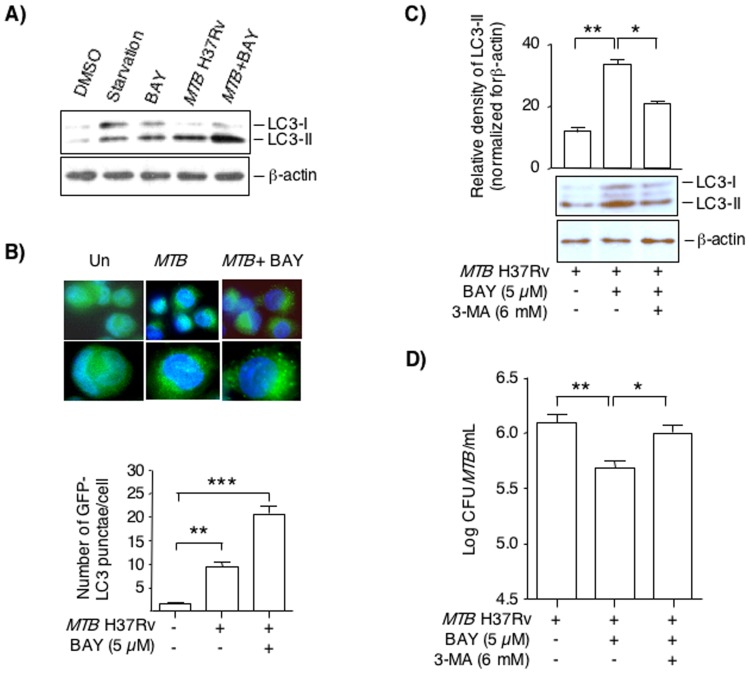
Inhibition of NFκB activation induces autophagy in THP-1 cells. (**A**) Control THP-1 cells (0.1% DMSO) and THP-1 cells subjected to serum starvation, 5 µM BAY, *MTB* infection, or both *MTB*+BAY for 24 hrs, followed by immunoblotting of nuclear-free whole cell lysates for LC3 and β-actin. A representative immunoblot of three independent experiments is shown. (**B**) Human THP-1 cells were transduced with lentivirus-GFP-LC3 and differentiated into macrophages, followed by infection with *MTB* for 24 hrs in the absence or presence of 5 µM BAY. The cells were fixed and stained with DAPI to visualize the nuclei (blue) and the number of GFP-positive punctae were quantified. *Upper panel*, representative immunofluorescence images of three independent experiments; *lower panel*, average number of GFP-LC3 punctae per cell. The data shown represent the mean ± SEM of duplicate wells/condition from three independent experiments. (**C**) *MTB*-infected THP-1 cells treated with BAY were incubated with or without 3-MA, an inhibitor of the early phase of the autophagic pathway. After 48 hrs, the cells were lysed and nuclear-free whole cell lysates (20 µg per lane) were separated by SDS-PAGE and immunoblotted for LC3-I, LC3-II and β-actin. The bar graph represents the relative densities of the LC3-II bands normalized for their corresponding β-actin bands for two independent experiments. (**D**) THP-1 cells were infected with *MTB* H37Rv alone, *MTB*+5 µM BAY, or *MTB*+BAY+6 mM 3-MA for 4 days and cell-associated *MTB* was quantified. Data shown are mean ± SEM from two independent experiments performed in duplicates. *p<0.05, **p<0.01, ***p<0.001.

THP-1 cells were exposed to the following conditions for 24 hrs: RPMI medium+10% FBS+1% glutamine+0.1% DMSO (unstimulated control), RPMI without FBS (starvation control), 5 µM BAY, *MTB*, or both *MTB* and BAY, followed by western blotting for LC3-I and LC3-II. This time point were found to be optimal to observe an increase in LC3-II by autophagy induction. Immunoblot showed increased LC3-II in lysates of starved cells compared to unstimulated control cells (DMSO) (**Figure 6A**). BAY treatment or *MTB* infection also modestly induced more LC3-II formation and when the cells were incubated with both *MTB* and BAY, there was a further increase in LC3-II (**Figure 6A**).

To validate the immunoblot data, we quantified the average number of LC3-II-positive autophagosomes per cell using THP-1 cells transduced with GFP-LC3 lentivirus. Transduced THP-1 cells were either left uninfected or infected with *MTB* for 24 hrs. We found that *MTB* infection alone significantly increased the number of GFP-LC3 punctae per cell compared to uninfected cells by 4 to 5-fold (**Figure 6B**). Following NFκB inhibition of *MTB*-infected THP-1 cells, the average number of GFP-LC3 punctae per cell further increased by an *additional* ∼2.5-fold (**Figure 6B**).

To corroborate whether autophagy plays a role in reducing the number of viable intracellular *MTB* recovered, we inhibited autophagy in *MTB*-infected THP-1 cells that were treated with BAY using the established autophagy inhibitor 3-methyladenine (3-MA), an inhibitor of class III PI3 kinase, an upstream signaling molecule of the autophagic signaling cascade [54]. As shown in **Figure 6C**, LC3-II was increased in *MTB*-infected THP-1 cells treated with BAY compared to THP-1 cells infected with *MTB* alone. With addition of 6 mM 3-MA in the culture medium, there was significant reduction in LC3-II levels. THP-1 cells were then infected with *MTB* alone or *MTB*+5 µM BAY with and without addition of 6 mM 3-MA. The cells were then cultured for 4 days and cell-associated *MTB* was quantified. As shown in **Figure 6D**, BAY reduced the number of *MTB* recovered, which was significantly abrogated by the addition of 3-MA.

### The effects of NFκB inhibition on intracellular *MTB* are independent of TNFα and IFNγ expression

**Figure 7:**
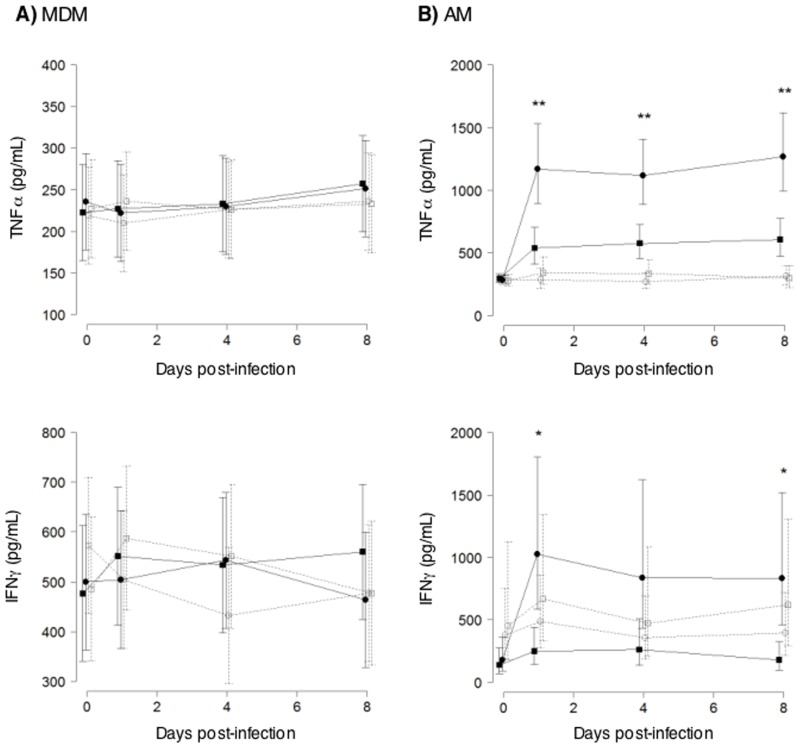
TNFα and IFNγ levels in MDM and AM infected with *MTB* H37Rv with or without NFκB inhibition. Primary human (**A**) MDM or (**B**) AM were infected with *MTB* H37Rv and after 1 hr, 24 hrs, 4 days, or 8 days of infection, supernatants were assayed for TNFα by ELISA and IFNγ by electrochemiluminescence. Data shown are estimated means with standard error bars from linear mixed model fits, based on seven independent experiments (MDM) or nine independent experiments (AM). *p<0.05 and **p<0.01 for cytokine expression in macrophages infected with *MTB* alone (closed circles) vs. *MTB*+BAY (closed squares). Control = (open circles) and BAY = (open squares).

TNFα can induce apoptosis [31], [55], [56] and IFNγ can induce autophagy in *MTB*-infected macrophages through immunity-related p47 guanosine triphosphatases (IRG) [6], [33]. Additionally, NFκB is an important transcription factor in the induction of both IFNγ and TNFα [57]. We examined the production of TNFα and IFNγ in *MTB*-infected human macrophages with or without NFκB inhibition. THP-1 cells had no detectable IFNγ and had minimal induction of TNFα by *MTB* at the time points examined (data not shown). In MDM, TNFα and IFNγ were minimally induced by *MTB* infection (**Figure 7**). In AM, TNFα and IFNγ were induced by *MTB* infection and BAY significantly inhibited their expression (**Figure 7**). The inhibition of TNFα and IFNγ expression in AM by BAY occurred despite concomitant decreases in *MTB* recovered. These results indicate that inhibition of bacterial growth following inhibition of NFκB activation is likely independent of TNFα and IFNγ production.

## Discussion

In human macrophages, inhibition of NFκB activation reduced the viability of intracellular *MTB* through increased induction of apoptosis and autophagy. While we showed that inhibition of NFκB enhanced macrophage response to *MTB*, it is important to note that NFκB is a ubiquitous transcription factor involved in many cellular processes associated with inflammation and infections. For example, suboptimal binding of NFκB to a *cis*-regulatory site on the 5′-flanking region of the *IFNγ* gene promoter leads to decreased production of IFNγ [58], a cytokine that is critically important in host-defense against mycobacterial infections. While others have shown that NFκB activation in mouse macrophages resulted in increased killing of mycobacteria, those studies primarily used non-pathogenic *Mycobacterium smegmatis* [59], [60]. These studies, combined with our findings, suggest that NFκB plays roles in both host-defense and host-susceptibility, depending on the microbial pathogen and the host species.

Since mice lacking the p50 subunit of NFκB suffered worsened *MTB* infection as compared to wildtype mice [23], we predicted that pharmacologic inhibition of NFκB activation in human macrophages would result in increased recovery of viable intracellular *MTB*. In contrast, we found that inhibition of NFκB activation resulted in significantly fewer viable intracellular *MTB* despite similar number of bacilli phagocytosed. One possible reason for the discrepancy between the prior mouse study and our current work with human macrophages is that production of nitric oxide, which is critically dependent on NFκB activation, plays a central role in killing intracellular *MTB* in mice [61]. Indeed, *MTB*-infected p50 –/– mice had significantly less inducible nitric oxide synthase expression in their lung tissues than infected wildtype mice [23]. In contrast, the role of nitric oxide in killing *MTB* in humans and human macrophages is considered far less important [62]. Additionally, there are inherent differences in the *in vivo* mouse study and our *in vitro*/*ex vivo* human macrophage studies. For example, in the p50-knockout mice, NFκB complexes containing the p50 subunit are silenced in all cell types including macrophages; in contrast, we examined macrophages in isolation. On the other hand, because we used a pharmacologic inhibitor of IKK as well as a dominant-negative IκBα, our experimental approach is expected to inhibit activation of not only p50-containing NFκB complexes but all NFκB complexes, which are comprised of dimers of various subunits (Rel A, c-Rel (p65), RelB, p50, and p52). Since there are several different types of NFκB complexes, an additional possibility is that there are differences between mouse and human cells in the ability of various NFκB complexes to affect immune response. This notion is consistent with the work of Gutierrez and co-workers who showed that in murine macrophages, activation of NFκB by some host lipids can induce killing of *MTB* whereas other lipids can induce killing of mycobacteria independent of NFκB activation [60]. Finally, the mouse study employed a different strain of *MTB* (Kurono strain) than the current study (H37Rv). Since different strains of *MTB* can induce varying degrees of apoptosis in host cells [51], differences in the *MTB* strain used may impact the importance of apoptosis in killing intracellular *MTB* with NFκB inhibition.

After discovering that inhibition of NFκB activation decreased intracellular *MTB* recovery, we next sought out potential mechanism(s). We found in all three types of human macrophages that decreased number of *MTB* recovered following NFκB inhibition correlated with increased apoptosis, an important effector killing mechanism of intracellular *MTB* [30], [48], [50], [51], [53]. Moreover, since there was non-uniformity of *MTB* infection of macrophages, apoptosis of relatively few but heavily infected cells could account for the significant reduction in the number of viable intracellular *MTB* recovered. While inhibition of classical apoptosis with inhibition of caspase-3 significantly abrogated the effects of BAY in terms of CFU and apoptosis, the reversal was not complete, suggesting that the effects of BAY may also involve caspase-3–independent programmed cell death mechanisms [63]. Based on our finding that the percent of non-viable cells as determined by Trypan blue staining was greater than the percentage of cells that were undergoing apoptosis, we believe that some of the infected cells are also undergoing necrosis as well. This is supported by studies showing that with MOI of 5 and 10, *MTB* H37Rv caused both apoptosis and necrosis of human macrophages [64]. This may explain the finding that even though inhibition of NFκB decreased the number of cell-associated *MTB*, the growth curve was still increasing over time; *i.e.*, we posit that in contrast to cells undergoing apoptosis, *MTB* remain viable in necrotic cells and upon release, have the potential to be phagocytosed by other macrophages and proliferate. Thus, we believe the overall fate of *MTB* cultured in macrophages is a dynamic process with some bacteria being killed while others proliferate, with the net biological effect being decreased number of intracellular *MTB* with inhibition of NFκB.

While TNFα has been shown by others to induce apoptosis in the context of *MTB* infection, there was little induction of TNFα in either THP-1 cells or MDM infected with *MTB*. For the THP-1 cells, we believe the minimal TNFα production may be due to the development of “tolerance” of these cells to subsequent *MTB*-induced cytokine production since we incubated the THP-1 cells with PMA for 2 to 3 days before changing the medium and infecting with *MTB*. We have since found that differentiating these cells over a shorter, overnight PMA incubation preserves the ability of THP-1 cells to produce cytokines in response to *MTB* without impact on intracellular survival of *MTB* [35].

Dhiman and co-workers previously showed that THP-1 cells, in which NFκB was suppressed via transfection with a mutated, dominant-negative form of IκBα, were less burdened by intracellular *MTB* [65]. Similar to our findings, the decrease in bacterial growth due to suppression of NFκB coincided with increased apoptosis of the transfected THP-1 cells [65]. We extended these findings by utilizing two types of primary human macrophages. Additionally, we showed that inhibition of classical apoptosis abrogated the reduction in intracellular CFU induced by NFκB inhibition. While Loeuillet *et al* showed that TLR2-mediated activation of NFκB prevented *MTB* infected THP-1 cells from undergoing Fas ligand mediated apoptosis [66], the anti-apoptotic effect of NFκB may involve induction of several anti-apoptotic gene products that ultimately inhibit caspase-3 activation [28]. NFκB may also interfere with apoptosis via direct protein-protein interaction such as direct coupling of NFκB subunits and c-IAP2, providing a signal amplification loop that promotes cell survival independent of de novo protein synthesis [28]. Activation of NFκB in the context of infections with *Bartonella*, *Ehrlichia*, or *Rickettsia* has also been shown to inhibit apoptosis of host cells by preventing the release of cytochrome *c* [67]. The increased vulnerability of AIDS patients to TB is most likely related to impaired effector T cell function [68], [69]. However, increased NFκB activation seen in HIV positive individuals could also impair the ability of their *MTB*-infected macrophages to undergo apoptosis [56], [70], providing another mechanism for their predisposition to TB.

**Figure 8:**
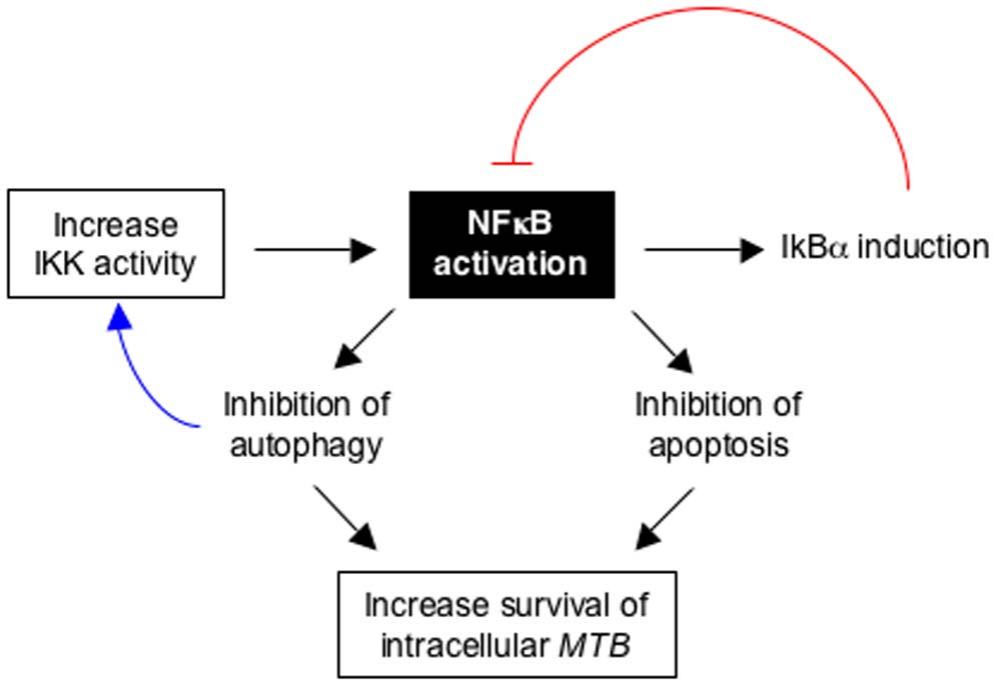
Diagram of the mechanisms by which NFκB activation promotes the intracellular survival of *MTB*. Based on our experimental findings, NFκB activation enhanced the intracellular survival of *MTB* through inhibition of apoptosis and autophagy in infected macrophages. Since NFκB can also induce the production of its inhibiting molecule IκBα (blue line) and NFκB inhibition of autophagy could potentially prevent degradation of IKK (red line), the ultimate effect of NFκB on survival of intracellular *MTB* in macrophages is likely a complex process. IKK = IκBα kinase.

NFκB inhibition also increased the formation of autophagosomes. Since autophagy has been shown to be an efficient killing mechanism of intracellular *MTB* [4], [6], [52], induction of apoptosis and autophagy are two mechanisms by which NFκB inhibition reduced the number of intracellular bacilli (**Figure 8**). Due to experimental limitations, we could not determine whether apoptotic cells were the *exact* same cells undergoing autophagy. However, it is plausible that autophagy may be activated in dying cells, perhaps as an attempt by dying cells to survive [71]. Since we detected increased autophagy in *MTB*-infected cells in which NFκB activation was inhibited at 24 hrs, whereas induction of apoptosis was seen later, it suggests that autophagy is not likely to be a secondary response to apoptosis in our experimental model. However, we cannot exclude the possibility that autophagy may be induced in response to the earliest stages of apoptosis. Consequently, there is a possibility that both processes may occur in the *same* cell over an extended period of time, but experimentally this would be very difficult to prove. Furthermore, the interplay between these NFκB-mediated mechanisms of *MTB* killing is likely complicated by the fact that NFκB can regulate its own activation by opposing mechanisms; *i.e.*, NFκB activation induces the production of its inhibitory molecule IκBα (**Figure 8** **, blue arrow**) and yet NFκB inhibition of autophagy can increase IKK activity since this kinase is normally degraded by the autophagic process [72] (**Figure 8** **, red line)**.

Cytokines and microbial products can activate other transcription factors such as AP-1 and ATF-2, which likely play important roles in the host immune response to *MTB* [42], [73]. A class of transcription factors known as nuclear receptors have recently been implicated in host-mycobacterial interactions [74], [75]. Mahajan and co-workers [75] showed that during *MTB* infection of macrophages, *MTB*-derived lipids and macrophage-derived lipids can combine with lipid-sensing nuclear receptors – peroxisome proliferator-activated receptor gamma (PPARγ) and testicular receptor 4 (TR4) – to induce expression of genes that ultimately enhances intracellular survival of *MTB*. In addition, activation of either of these receptors induced alternate activation of macrophages to the M2 phenotype [75], which would be expected to impair effective host immune response against *MTB*. These nuclear receptors are immune evasive factors since mycobacteria can induce the expression of PPARγ [74]. In contrast, activation of another type of nuclear receptor (LXRα) decreases lipidogenesis and enhances host immunity against *MTB* [75].

In summary, we found that inhibiting NFκB activation in macrophages resulted in increased apoptosis and autophagy, and decreased recovery of viable intracellular *MTB*. There are hundreds of natural and synthetic compounds known to inhibit NFκB activation, including various antioxidants, proteasome and protease inhibitors, and IKK inhibitors (http://people.bu.edu/gilmore/nf-kb/inhibitors/index.html). It is clear that the role of NFκB following *MTB* infection is complicated. Future studies could consider utilizing mixed cell cultures to determine the effects of NFκB inhibition on the collaboration between macrophages and T cells. Furthermore, it is plausible that NFκB activation may be important in the early phase of infection but continued activation may be deleterious to the host. Thus, future *in vivo* studies could inhibit NFκB at different time points of infection to begin to delineate “host-protective” vs. “host-susceptible” NFκB-mediated pathways.

## Supporting Information

Figure S1 **BAY 11-7082 (BAY) does not affect** ***MTB H37Rv*** **growth in Middlebrook 7H9 medium**. *MTB* H37Rv (2.4×10^5^ bacilli/mL) was incubated in 7H9 liquid medium containing 0.1% (v/v) DMSO vehicle (control) or 5 µM or 10 µM BAY for 4 and 8 days, and CFU determined. Data shown are the mean ± SEM of two independent experiments performed in duplicates.
(TIF)

Figure S2 **Cytotoxicity of** ***MTB*** **-infected THP-1 cells with and without BAY treatment**. The percentages of dead cells were determined by trypan blue dye exclusion after 5 days of infection. Data are the means ± SEM from two independent experiments performed in duplicates. **p<0.01.
(TIF)
