# Supplementary figures and images for: Inhibition of Nuclear Factor-Kappa B Activation Decreases Survival of Mycobacterium tuberculosis in Human Macrophages
Source: PLoS One. 2013 Apr 25;8(4):e61925. doi: 10.1371/journal.pone.0061925 (PMC3636238; doi:10.1371/journal.pone.0061925)

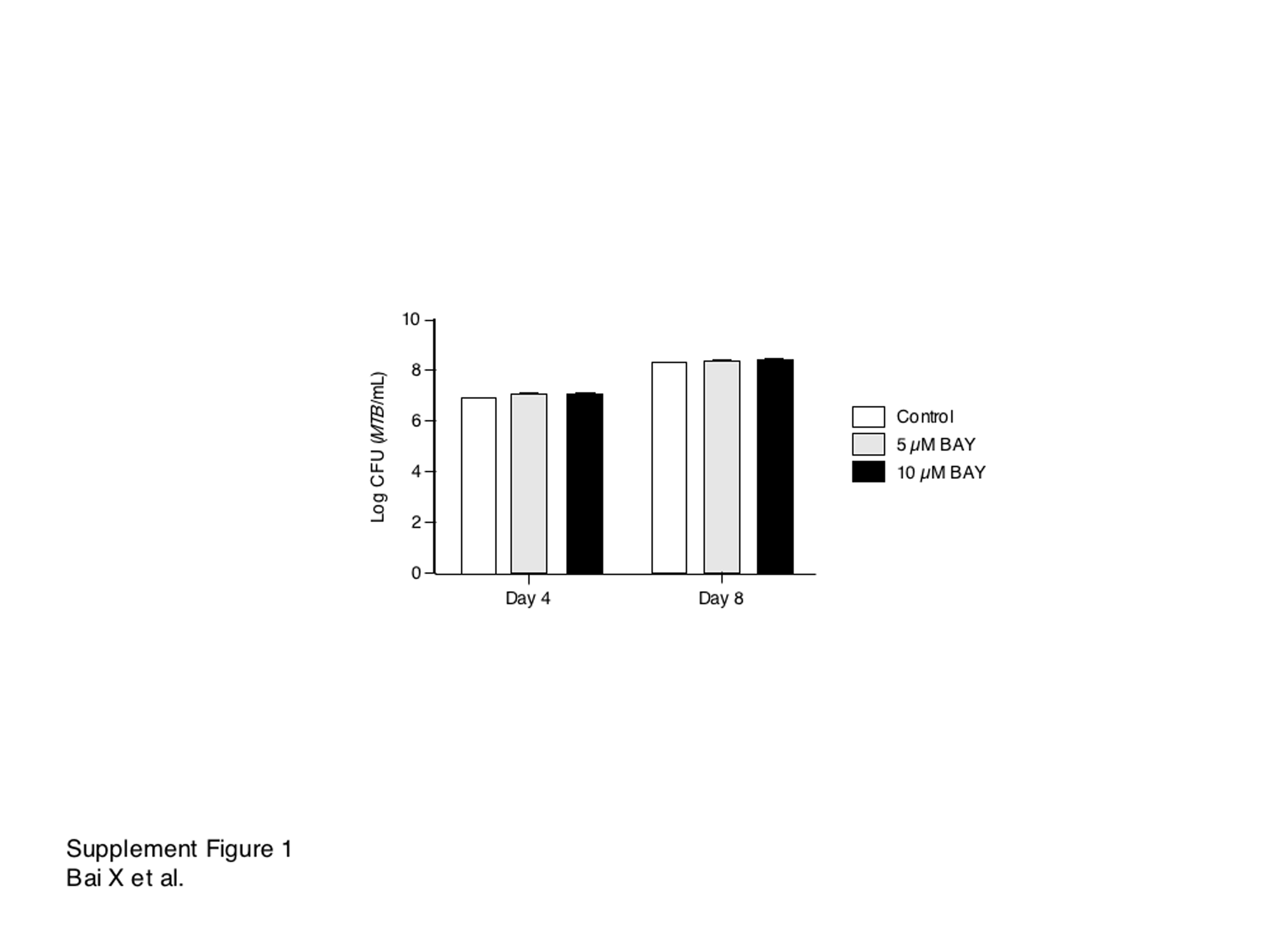

Supplement: Figure S1 — BAY 11-7082 (BAY) does not affect MTB H37Rv growth in Middlebrook 7H9 medium. MTB H37Rv (2.4×10^5^ bacilli/mL) was incubated in 7H9 liquid medium containing 0.1% (v/v) DMSO vehicle (control) or 5 µM or 10 µM BAY for 4 and 8 days, and CFU determined. Data shown are the mean ± SEM of two independent experiments performed in duplicates. (TIF) [file pone.0061925.s001.tif]

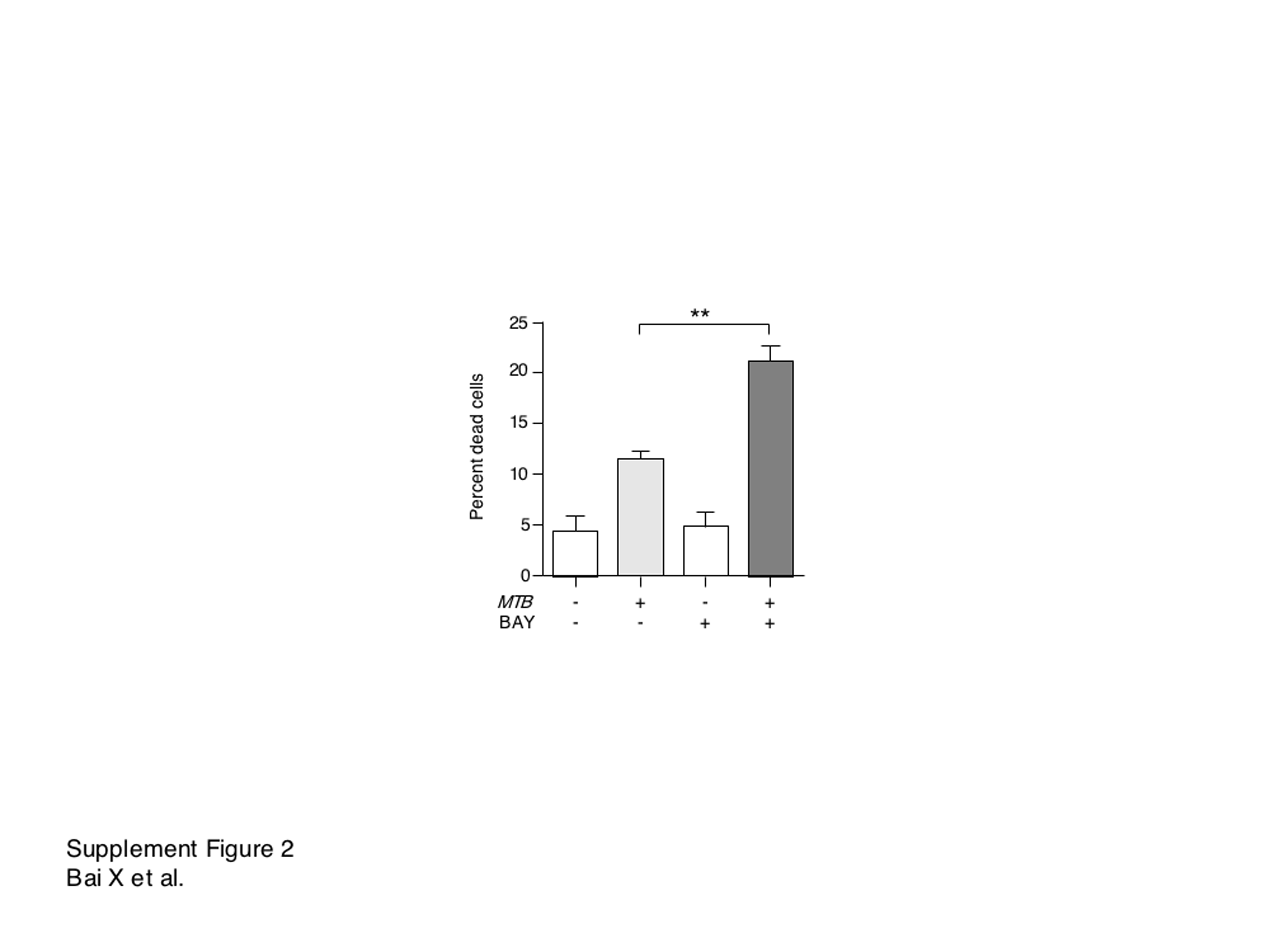

Supplement: Figure S2 — Cytotoxicity of MTB -infected THP-1 cells with and without BAY treatment. The percentages of dead cells were determined by trypan blue dye exclusion after 5 days of infection. Data are the means ± SEM from two independent experiments performed in duplicates. **p<0.01. (TIF) [file pone.0061925.s002.tif]
